# Characterization of a novel *Lactobacillus *species closely related to *Lactobacillus johnsonii *using a combination of molecular and comparative genomics methods

**DOI:** 10.1186/1471-2164-11-504

**Published:** 2010-09-17

**Authors:** Luz-Adriana Sarmiento-Rubiano, Bernard Berger, Déborah Moine, Manuel Zúñiga, Gaspar Pérez-Martínez, María J Yebra

**Affiliations:** 1 Laboratorio de Bacterias Lácticas y Probióticos. IATA-CSIC, P.O. Box 73, 46100 Burjassot, Valencia, Spain; 2 Nestlé Research Center, CH-1000 Lausanne 26, Vers-chez-les-Blanc, Switzerland

## Abstract

**Background:**

Comparative genomic hybridization (CGH) constitutes a powerful tool for identification and characterization of bacterial strains. In this study we have applied this technique for the characterization of a number of *Lactobacillus *strains isolated from the intestinal content of rats fed with a diet supplemented with sorbitol.

**Results:**

Phylogenetic analysis based on 16S rRNA gene, *recA*, *pheS*, *pyrG *and *tuf *sequences identified five bacterial strains isolated from the intestinal content of rats as belonging to the recently described *Lactobacillus taiwanensis *species. DNA-DNA hybridization experiments confirmed that these five strains are distinct but closely related to *Lactobacillus johnsonii *and *Lactobacillus gasseri*. A whole genome DNA microarray designed for the probiotic *L. johnsonii *strain NCC533 was used for CGH analysis of *L. johnsonii *ATCC 33200^T^, *L. johnsonii *BL261, *L. gasseri *ATCC 33323^T ^and *L. taiwanensis *BL263. In these experiments, the fluorescence ratio distributions obtained with *L. taiwanensis *and *L. gasseri *showed characteristic inter-species profiles. The percentage of conserved *L. johnsonii *NCC533 genes was about 83% in the *L. johnsonii *strains comparisons and decreased to 51% and 47% for *L. taiwanensis *and *L. gasseri*, respectively. These results confirmed the separate status of *L. taiwanensis *from *L. johnsonii *at the level of species, and also that *L. taiwanensis *is closer to *L. johnsonii *than *L. gasseri *is to *L. johnsonii*.

**Conclusion:**

Conventional taxonomic analyses and microarray-based CGH analysis have been used for the identification and characterization of the newly species *L. taiwanensis*. The microarray-based CGH technology has been shown as a remarkable tool for the identification and fine discrimination between phylogenetically close species, and additionally provided insight into the adaptation of the strain *L. taiwanensis *BL263 to its ecological niche.

## Background

A number of species belonging to the genus *Lactobacillus *are indigenous to the gastrointestinal tract of humans and animals [1-3]. Due to their ecological and commercial interest, substantial efforts have been concentrated in the past years to isolate and identify new strains of lactobacilli. Many of those strains belong to the *Lactobacillus acidophilus *complex. The first major study on this group using DNA-DNA hybridization defined two main homology groups, A and B, with six subgroups [4], that later became distinct species [5]. Strains of the group A were classified as the species *L. acidophilus*, *Lactobacillus amylovorus*, *Lactobacillus crispatus *and *Lactobacillus gallinarum*, and strains of the group B became *Lactobacillus gasseri *and *Lactobacillus johnsonii *[5]. Some strains of *L. acidophilus *and *L. johnsonii *are commercialized as probiotics and they have been subjected to extensive studies aimed to infer the mechanisms governing their beneficial health effects. *L. acidophilus *strain NCFM increased the immunogenicity of an oral human rotavirus vaccine in animals [6]. *L. acidophilus *strain Bar13 interfered with the adhesion of enteropathogenic bacteria to enterocytes and inhibited IL-8 production by HT-29 cells, suggesting a potential to protect intestinal cells from acute inflammatory response [7]. *L. johnsonii *strain La1 (NCC533) has been shown to suppress gene expression of proinflammatory cytokines, supposedly involved in atopic dermatitis [8,9] and acts as an immunomodulator [10-12]. The genome of *L. johnsonii *strain NCC533 has been sequenced [13] and has enabled a number of studies aimed to determine the genes which are expressed *in vivo *and those whose expression is specifically necessary for long persistence in the gut [14,15].

The bacterial species concept has been widely discussed [16,17], but no consensus has been reached to establish a regularly acceptable definition. In the case of prokaryotic pathogens, species are traditionally identified on the basis of the disease they caused, regardless of genetic considerations. However, for the vast majority of bacteria, phenotypic characteristics are generally not as precise as identification based on genotypic methods. The observation that there are a fraction of genes found in bacterial genomes shared by all members of a species and a fraction present only in a subset of the population gave rise to the core genomic hypothesis [18]. It postulates that there is a core of genes responsible for keeping a species identity and auxiliary genes responsible for gene transfer and adaptation of strains to the environment. The incorporation of molecular data permitted to state that a bacterial species is "a category that circumscribes a (preferably) genomically coherent group of individual isolates/strains sharing a high degree of similarity in (many) independent features, comparatively tested under highly standardised conditions" [19]. Essentially, a species would be a group of strains with certain level of phenotypic consistency and showing DNA-DNA re-association rates greater than 70% [19]. However, this variability cut-off method is not always appropriate due to the fact that different clearly recognized species have a large range of genetic variation. Since DNA sequencing is nowadays an accessible technique to most laboratories, comparative sequence analysis of the 16S rRNA gene has been extensively used for species identification in novel isolates [20]. The *ad hoc *committee for the re-evaluation of the species definition in bacteriology considered sequencing of housekeeping genes, DNA profiling and DNA arrays as very valuable methods for species delineation and phylogenetic positioning [19].

The *L. acidophilus *group constitutes a paradigm of a compact cluster showing closely related species that offers special difficulties for the correct taxonomic allocation of new strains. Several techniques have been applied to identify members of the *L. acidophilus *group, such as 23S-rRNA-targeted oligonucleotide probe hybridization [21], random amplification of polymorphic DNA [22,23], sequence alignment of the V1 region of the 16S rRNA encoding gene [24] and automated ribotyping [25]. Recently, more complex genomic techniques based on multilocus sequence analysis of five housekeeping genes and comparative genome hybridization (CGH) using microarrays have been successfully applied to the *L. acidophilus *complex [26]. They showed that *L. johnsonii*, *L. gasseri *and *L. acidophilus *constitute three clearly independent and consistent genomic groups, although *L. gasseri *was more closely related to *L. johnsonii *than to *L. acidophilus*. The bacterial genomic variability between strains belonging to the same species or between closely related species comes from mobile DNA elements and from variable regions. The latest may be required for environmental adaptation and could be acquired by lateral gene transfer. The *L. johnsonii *NCC533 chromosome harbors three regions that contain two prophages, Lj928 and Lj965, and a 6-kb potentially autonomous unit [27,28]. These genetic elements represent more than half of the identified strain-specific DNA and have been extensively studied [26-28]. *L. johnsonii *NCC533 also carries four further integrases and several IS elements flanking or disrupting diversity regions [26-28].

A previous work studying the influence of sorbitol intake on the population of rat intestinal lactobacilli showed the presence of five species: *L. johnsonii*, *Lactobacillus intestinalis*, *Lactobacillus murinus*, *Lactobacillus reuteri *and *Lactobacillus *sp. BL263 (previously named as AD102) [29]. Sorbitol ingestion resulted in an increment of *L. reuteri *and *Lactobacillus *sp. BL263 cell numbers [29]. Although strain *Lactobacillus *sp. BL263 could be assigned to the *L. acidophilus *group, its species allocation was still uncertain. In the present study, standard taxonomic methods and microarray-based CGH analysis have been implemented for the identification and characterization of *Lactobacillus *sp. BL263 and four additional rat intestinal isolates (BL301, BL302, BL303 and BL304) displaying identical PCR-DGGE pattern.

## Methods

### Bacterial strains, culture conditions, DNA extraction

The *Lactobacillus *strains used in this work are listed in Table 1 and they were grown in MRS medium (Difco) at 37°C during 48 h under static conditions. Chromosomal DNA was isolated as described before [30].

**Table 1:** List of strains used in this study

Species	Strain^a^	Culture collection^b^	Host
*Lactobacillus acidophilus*	BL17	CECT 903^T^	human
*Lactobacillus acidophilus*	BL279	CECT 4529	chicken intestine
*Lactobacillus acidophilus*	BL280	CECT 4179	rat feces
*Lactobacillus crispatus*	BL221		human feces
*Lactobacillus crispatus*	BL278	DSMZ 20584^T^	human eye
*Lactobacillus gasseri*	BL223		human feces
*Lactobacillus gasseri*	BL277	ATCC 33323^T^	human
*Lactobacillus gasseri*	BL292	NCC 2858	unknown
*Lactobacillus gasseri*	BL294	NCC 2856	unknown
*Lactobacillus gasseri*	BL296	NCC 2857	unknown
*Lactobacillus intestinalis*	BL260		rat intestine
*Lactobacillus intestinalis*	BL288	DSMZ 6629^T^	rat intestine
*Lactobacillus johnsonii*	BL261		rat intestine
*Lactobacillus johnsonii*	BL281	CECT 289	unknown
*Lactobacillus johnsonii*	BL287	ATCC 33200^T^	human blood
*Lactobacillus johnsonii*	BL295	NCC 2822	dog feces
*Lactobacillus johnsonii*	BL297	NCC 1741	unknown
*Lactobacillus johnsonii*	BL298	NCC 533	human feces
*Lactobacillus johnsonii*	BL299	NCC 2767	dog feces
*Lactobacillus murinus*	BL262		rat intestine
*Lactobacillus reuteri*	BL259		rat intestine
*Lactobacillus taiwanensis*	BL263	CECT 7394	rat intestine
*Lactobacillus taiwanensis*	BL301		rat intestine
*Lactobacillus taiwanensis*	BL302		rat intestine
*Lactobacillus taiwanensis*	BL303		rat intestine
*Lactobacillus taiwanensis*	BL304		rat intestine
*Lactobacillus taiwanensis*	BL340	DSMZ 21401^T^	silage

^a^BL, Culture Collection from our laboratory. Strains BL263, BL260, BL261, BL262 and BL259 were previously named as AD102, AD38, AD99, AD100 and AD23, respectively [29].

^b^CECT, The Spanish Type Culture Collection; DSMZ, German Collection of Microorganisms and Cell Cultures; NCC, Nestlé Culture Collection; ATCC, American Type Culture Collection.

### 16S rRNA encoding gene and multilocus sequence analyses

The housekeeping genes selected for multilocus sequence analysis based on the results of previous studies on *Lactobacillus *species [26,31] were recombinase A (*recA*), phenylalanyl-tRNA synthase (*pheS*), CTP synthetase (*pyrG*) and translational elongation factor Tu (*tuf*). PCR reactions were performed with the Expand High Fidelity PCR System (Roche), using chromosomal DNA and the primers listed in Table 2. DNA sequencing was performed by the Central Service of Research Support of the University of Valencia (Spain) by using the Dideoxynucleotide DNA chain termination method. Both strands of the PCR fragments were sequenced. In the case of the *recA *gene, the PCR fragments were cloned in *Escherichia coli *using the pMOS Blue vector contained in the Blunt- ended cloning kit (GE Healthcare) following the manufacturer instructions. Plasmid DNA was isolated from each clone containing a portion of the *recA *gene from the *Lactobacillus *strains studied here. The partial *recA *genes were sequenced using the M13 universal and reverse primers.

**Table 2:** Multilocus sequence analysis

Gene	Primer	Nucleotide sequence	Amplified fragment size (bp)	Number of alleles in *L. taiwanensis strains*
16S rRNA	16S-27 for	5'-AGAGTTTGATCCTGGCTCAG	1552	1
	16S-1552rev	5'-AAGGAGGTGWTCARCCGCA		
				
*recA*	RecAfor	5'-GAAAARRAYTTYGGWAARGGYKCDRTBATGCG	740	0
	RecArev	5'-TACATRATRTCDACTTCWSMNMSYTTRAATGG		
				
*pheS*	PheSfor	5'-KGGDCGYAAGGGTGAATTAAC	908	0
	PheSrev	5'-ACATCRTTWGTRTAGAARTCACGAATATC		
				
*tufF*	Tuf-for	5'-ATGGCAGAAAAAGAACATTACG	1176	1
	Tuf-rev	5'-AGTAACYTGACCRGCACCAAC		
				
*pyrG*	PyrGfor	5'-TTATGTTACYGAYGATGGTAC	908	0
	PyrGrev	5'-ACCACGWGTACCAAAACCAC		

### Phylogenetic analysis

The sequences were either obtained in this work, retrieved from the GenBank database or from the Ribosomal Database Project II (RDP) [32]. Multiple alignments were obtained using ClustalX [33]. Positions of doubtful homology and gaps were removed by using Gblocks [34]. The phylogenetic reconstruction was performed by maximum likelihood as implemented in PHYML [35] with the GTR, substitution model in combination with estimation of the transtition/transversion ratio by maximizing the likelihood of the phylogeny, and estimations from the data set of the proportion of invariants and the shape parameter (alpha) of the gamma distribution to account for substitution rate heterogeneity among sites. Bootstrap support values were obtained through the analysis of 500 pseudoreplicates.

### Physiological characterization

Carbohydrate fermentation patterns were determined by using the API 50 CH (BioMérieux, S.A.) according to the supplier instructions. Results were recorded after 48 h at 37°C. Growth at 15°C, 45°C, pH 4.5 or in the presence of NaCl (4.5% and 7.0%) was determined in MRS broth (Difco). All the growth analyses were done at least in duplicate. Data were analysed using Pearson's correlation coefficient (XLSTAT programme). A tree was created by cluster analysis using the unweighted pair-group method with arithmetic averages (UPGMA).

### PAGE analysis of whole-cell protein

Cultures were grown in 10 ml MRS at 37°C to an OD_550 _of 1.0. Cells were collected by centrifugation, washed with Tris-HCl 100 mM, pH 7.5, resuspended in 1 ml of the same buffer plus sucrose 0.5 M and lysozyme 2.5 mg/ml. After incubation for 1 h at 37°C, cells were washed, collected by centrifugation and resuspended in 50 μl of SDS-PAGE loading buffer. Cells were lysed at 100°C 5 min and 20 μl of total extract were separated on a 12% SDS-PAGE using standard protocols [36]. The gels were stained with Coomassie brilliant blue. For each strain among two and four independent extracts were performed and analysed by SDS-PAGE. A PAGE analysis representative of each strain is shown in the results. Data were analysed using Pearson's correlation coefficient (BioNumerics 4.6 Applied Maths Kortrijk). A tree was created by cluster analysis using UPGMA.

### DNA-DNA hybridization assays

DNA macro-arrays on nylon membranes (Hybond N, Amersham Biosciences) were performed by using a dot blot assay according to Hänninen et al. [37] with some modifications. Three aliquots (160, 80 and 40 ng) of denatured chromosomal DNA samples of 26 *Lactobacillus *strains were spotted on duplicated membranes. Six digoxigenin-labelled probes were prepared by random priming using the DIG DNA Labelling kit (Roche) and genomic DNA isolated from the type strains of *L. intestinalis*, *L. johnsonii*, *L. crispatus*, *L. gasseri*, *L. acidophilus *or *L. taiwanensis*. Those probes were hybridized against six identical macroarrays with 26 *Lactobacillus *strains. Hybridization, washing and staining was carried out as recommended by the manufacturer using the CDP-*Star *chemiluminescent substrate (Roche). Chemiluminescence was detected in a Fujifilm LAS 1000 imaging system (Fuji Photo Film Co. Ltd.) and it was measured using the Image Gauge Version 4.0 programme. The DNA-DNA hybridization experiments were done in triplicate.

### Comparative genomic hybridization on microarrays

Based on the genome sequence of *L. johnsonii *NCC533 [13], Agilent 60-mer oligo microarrays were designed with 5 to 6 probes spreading the coding sequence of each gene (Agilent technologies Inc., USA). Genomic DNA was prepared and labelled as previously described [26]. Hybridization reactions were performed in a volume of 210 μl with 10 μl of labelled DNA, 70 μl of nuclease free water, 25 μl of control target Agilent and 105 μl of Agilent hybridization buffer, following the recommendations of the manufacturer. The slides were washed 10 min at RT in 6× SSC, 0.005% Triton x-100 and 10 min on ice in 0.1× SSC, 0.005% Triton x-100. The slides were immediately dried by centrifugation and scanned at 10 μm with a Scanarray 4000 (Packard Biochip Technologies, Billerica, MA, USA). Data were extracted with Imagene 5.6 (Biodiscovery, El Segundo, CA, USA) and treated with homemade scripts in Python language http://www.python.org. Probes were eliminated from the analysis if their signal strength was lower than twice the standard deviation of the local background when hybridized with *L. johnsonii *NCC533 genomic DNA. The signal ratio (Cy3-labeled unknown strain DNA versus Cy5-labeled *L. johnsonii *NCC533 DNA) of each spot was calculated without background subtraction and normalized based on our previous experience of CGH [26]. Having 5 to 6 probes per gene, each gene signal ratio was given by the median of the corresponding probes values. Considering the low level of strain variability for the tested molecule (DNA), the large number of probes per gene and the robustness of the median, biological replicates of hybridizations focused only on the most critical analysis: self-NCC533 (validation of the arrays), ATCC33200^T^/NCC533 (intra-species comparison), and BL263/NCC533 (inter-species comparison with the novel *Lactobacillus*).

### Accession numbers

The 16S rRNA gene, *recA*, *pheS*, *tuf *and *pyrG *sequences generated in this study were deposited in the GenBank under accession numbers FJ556999 to FJ557013, FJ557014 to FJ557023, GU121623 to GU121628 and HM777006, GU121629 to GU121634 and HM777008, and GU121635 to 121640 and HM777007, respectively. The microarrays data have been deposited in NCBI's Gene Expression Omnibus [38] and are accessible through GEO Series accession number GSE21627.

## Results

### 16S rRNA encoding gene and multilocus sequence analyses positioned the novel *Lactobacillus *isolates within the *Lactobacillus acidophilus *group

**Figure 1:**
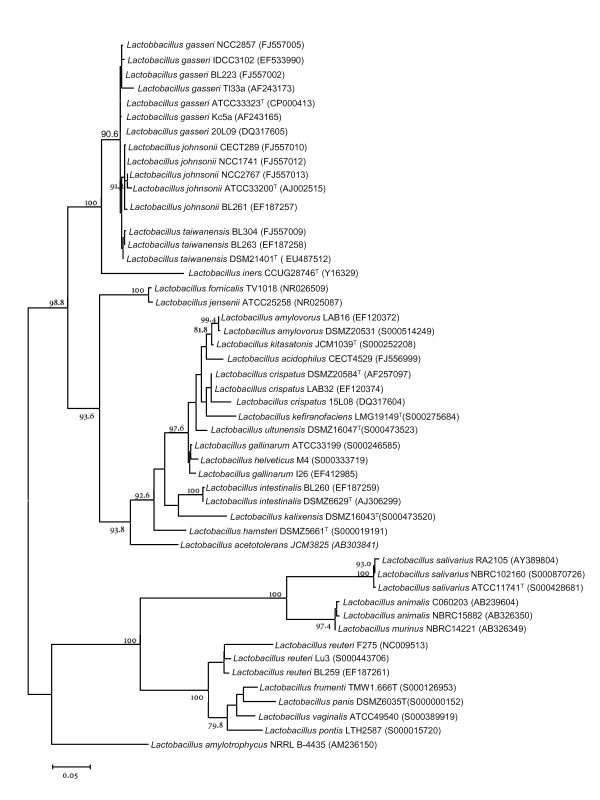
**Phylogenetic tree showing relationships among 16S rRNA gene sequences of species in the *Lactobacillus acidophilus *group, including *Lactobacillus taiwanensis*, and species representing different lineages within the genus *Lactobacillus***. The tree was created using a maximum-likelihood approach and numbers at branch points are bootstrap values (based on 500 samplings expressed in percentages). Only bootstrap values over 75% are shown. Bar indicates sequence divergence. The tree has been arbitrarily rooted. In parenthesis are shown the GenBank or Ribosomal Database Project II accession numbers.

Sequences of the 16S rRNA gene (approximately 1,440 bp) of the rat isolates *Lactobacillus sp*. BL263, BL301, BL302, BL303 and BL304 were determined. Sequences of BL263, BL301, BL302 and BL303 were identical (data not shown) and shared a 99.9% sequence identity with BL304 strain. A preliminary analysis showed that these sequences were very similar to sequences of *L. gasseri *and *L. johnsonii*. Subsequently, a phylogenetic tree was obtained by using the tools available in RDP [32]. On the basis of this reconstruction, sequences of species close to *L. gasseri *and *L. johnsonii *were selected for a detailed phylogenetic reconstruction by maximum likelihood. During the preparation of this manuscript, the novel *L. taiwanensis *species isolated from silage was described [39]. The phylogenetic reconstruction based on 16S rRNA gene sequences suggested that these strains belong to *L. taiwanensis *species and that they are closely related to *L. johnsonii *and *L. gasseri *(Fig. 1). The identity between the 16S rRNA gene of *L. taiwanensis *and *L. johnsonii *and *L. gasseri *strains ranged from 99.2% to 99.7% and 98.5% to 99.5%, respectively. This is higher than the generally accepted >97% identity threshold for the species definition, but this problem is known for long in the acidophilus group [24].

**Figure 2:**
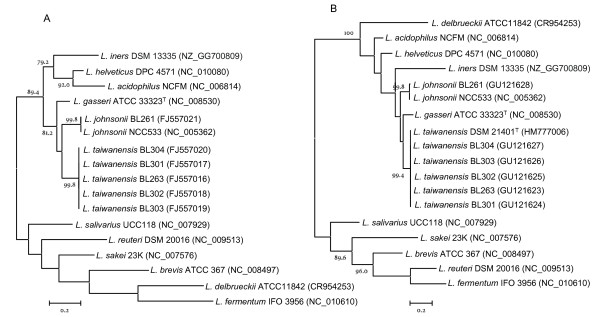
**Phylogenetic tree showing relationships among *recA *(A) and *pheS *(B) gene sequences of species in the *Lactobacillus acidophilus *group, including *Lactobacillus taiwanensis*, and species representing different lineages within the genus *Lactobacillus***. The trees were created using a maximum-likelihood approach and numbers at branch points are bootstrap values (based on 500 samplings expressed in percentages). Only bootstrap values over 75% are shown. Bar indicates sequence divergence. The trees have been arbitrarily rooted. In parenthesis are shown the GenBank accession numbers.

**Figure 3:**
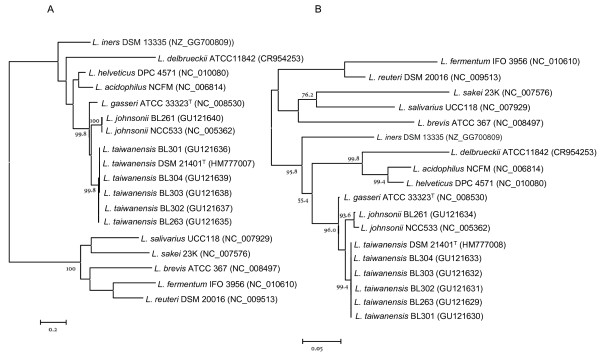
**Phylogenetic tree showing relationships among *pyrG *(A) and *tuf *(B) gene sequences of species in the *Lactobacillus acidophilus *group, including *Lactobacillus taiwanensis*, and species representing different lineages within the genus *Lactobacillus***. The trees were created using a maximum-likelihood approach and numbers at branch points are bootstrap values (based on 500 samplings expressed in percentages). Only bootstrap values over 75% are shown. Bar indicates sequence divergence. The trees have been arbitrarily rooted. In parenthesis are shown the GenBank accession numbers.

The analysis of the *recA*, *pheS*, *pyrG *and *tuf *gene sequences from strains BL263, BL301, BL302, BL303, BL304 and *L. taiwanensis *DSM 21401^T ^also led to phylogenetic trees that clearly separated a cluster encompassing *L. taiwanensis *and the rat intestinal isolates from all the *L. johnsonii *and *L. gasseri *strains as indicated by the bootstrap values (Fig. 2 and Fig. 3). Therefore, we concluded that our isolates belong to *L. taiwanensis*. These phylogenetic reconstructions were in general agreement with that obtained with the 16S rRNA gene sequences described above. Although highly conserved, those genes showed a higher degree of variability between related bacteria than the 16S rRNA gene. Therefore, the multilocus sequence analysis provided higher discriminating power which is more suited for phylogenetic and taxonomic reconstruction within the closely related species that constitute the *L. acidophilus *group.

### Phenotypic and protein profile analyses did not resolve species

**Figure 4:**
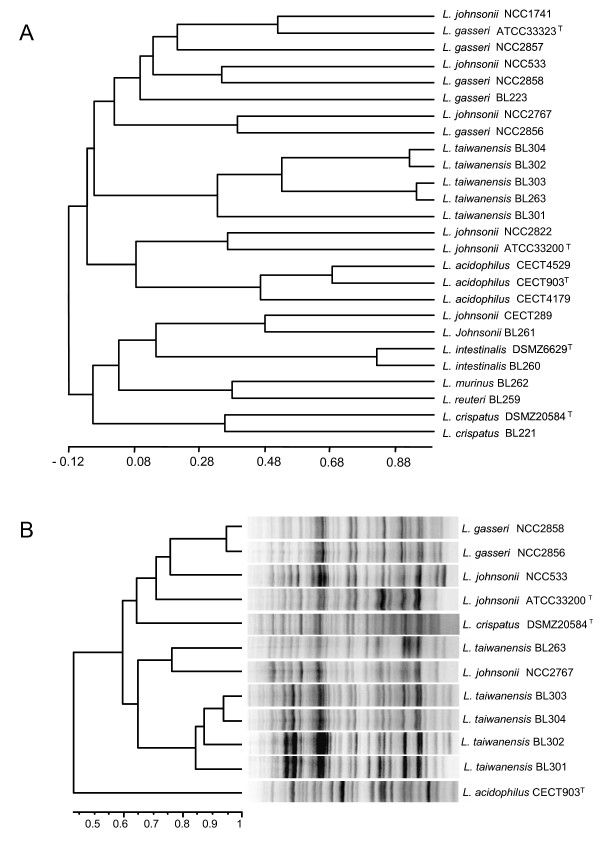
**Dendrogram derived from the analysis of phenotypic properties (A) and SDS-PAGE whole-cell protein profiles (B) of *Lactobacillus taiwanensis *strains and related strains of species from the genus *Lactobacillus***. The analysis was performed using Pearson´s correlation coefficient (*r*) and the values are shown at the bottom of each dendrogram. Both trees (A and B) were constructed using the unweighted pair-group method with arithmetic averages (UPGMA).

The carbohydrate fermentation patterns, and the growth profiles of 26 lactobacilli (including the novel isolates and members of the *L. acidophilus *group) at different temperatures and salt concentrations were used to construct a dendrogram (Table 3 and Fig. 4). Similarly, a comparative analysis of whole-cell protein patterns was performed with bacterial protein extracts of the novel isolates and other members of the *L. acidophilus *group (Fig. 4). Neither the phenotypic dendrogram, nor the cluster analysis of the SDS-PAGE protein profiles showed a good agreement with the phylogenetic relationships inferred from genetic markers. These phenotypic and protein profiles analyses agree with previous taxonomic analyses [40] that showed a lack of correlation between phylogenetic positioning and physiological/biochemical properties in lactobacilli. Notwithstanding, *L. taiwanensis *strains grouped together in a subcluster of the phenotypic analysis with an internal similarity above 30% (Fig. 4).

**Table 3:** Differential phenotypic features of *Lactobacillus taiwanensis *strains (BL263, BL301, BL302, BL303, BL304) with respect to related species of the genus *Lactobacillus *(*L. acidophilus, L. crispatus, L. gasseri, L. intestinalis, L. johnsonii, L. murinus *and *L. reuteri*)

Strains^a^	BL 263	BL 301	BL 302	BL 303	BL 304	BL 17	BL 279	BL 280	BL 221	BL 278	BL 223	BL 277	BL 292	BL 294	BL 296	BL 260	BL 288	BL 261	BL 281	BL 287	BL 295	BL 297	BL 298	BL 299	BL 262	BL 259
L-Arabinose	-	-	-	-	-	-	-	-	-	-	-	-	-	-	-	-	-	-	-	-	-	-	-	-	d	d
Ribose	-	-	-	-	-	-	-	-	-	-	-	-	-	-	-	-	-	-	-	-	-	-	-	-	d	d
D-Xylose	-	-	-	-	-	-	-	-	-	-	-	-	-	-	-	-	-	-	-	-	-	-	-	-	-	d
Galactose	-	-	-	-	d	-	+	+	+	-	+	+	-	+	-	-	+	d	d	+	d	-	-	d	+	+
D-Fructose	+	+	+	+	+	+	+	+	+	+	+	+	+	+	+	+	+	+	+	+	+	+	d	+	+	-
D-Mannose	+	+	+	+	+	+	+	+	d	-	+	+	-	+	+	+	+	+	d	+	+	+	d	+	+	-
Mannitol	-	-	-	-	-	-	-	-	+	-	-	-	-	-	-	d	+	-	-	-	-	-	-	-	+	-
α-methyl-D-Glucoside	-	-	-	-	-	-	-	d	d	-	-	-	-	-	-	-	-	-	-	-	-	-	-	-	-	-
N-Acethyl Glucosamine	-	d	+	-	+	+	+	+	+	+	+	+	+	+	+	+	+	d	d	+	+	+	+	+	+	-
Amygdalin	-	-	-	-	-	+	+	+	-	-	+	+	-	+	+	-	-	-	-	-	-	-	-	-	-	-
Arbutin	-	-	-	-	-	-	-	-	d	+	+	+	+	-	+	-	-	-	-	-	-	-	-	-	+	-
Aesculin	+	+	+	+	+	+	+	+	+	+	+	+	+	+	+	-	-	d	-	+	+	+	+	+	+	-
Salicin	-	-	-	-	-	+	+	d	d	+	+	+	d	+	+	-	-	-	-	-	d	d	-	-	+	-
Cellobiose	+	d	+	+	+	+	+	d	+	+	+	+	+	+	-	-	-	-	-	d	d	+	+	-	+	-
Maltose	+	+	+	+	+	+	+	+	+	+	+	+	d	+	+	+	+	+	+	+	+	+	-	+	+	+
D-Lactose	-	-	-	-	-	+	+	+	+	-	+	+	-	+	-	-	-	-	-	d	d	+	+	-	+	+
Melibiose	-	-	-	-	-	-	-	d	-	-	-	-	-	-	-	-	-	-	-	-	-	-	-	-	+	+
Trehalose	+	-	+	+	+	+	+	+	+	-	+	+	+	+	+	-	-	+	+	-	+	+	+	-	+	-
D-Raffinose	-	-	-	-	-	d	+	+	d	-	-	-	-	-	-	-	d	-	-	+	+	-	-	+	+	+
Starch	-	-	-	-	-	-	-	-	+	+	+	+	-	-	-	-	-	d	d	d	d	d	-	-	-	-
Glycogen	-	-	-	-	-	-	-	-	+	+	-	-	-	-	-	-	-	-	-	-	-	-	-	-	-	-
β-Gentibiose	d	-	+	+	+	+	+	+	d	-	+	+	+	+	+	-	-	+	-	+	+	+	-	+	+	-
D-Turanose	-	-	-	-	-	-	-	-	-	-	+	-	-	-	-	-	-	-	-	-	-	-	-	-	d	-
D-Tagatose	-	-	-	-	-	-	-	-	-	-	+	+	d	+	-	-	-	-	d	d	-	d	-	-	-	-
Growth 15°C	-	-	-	-	-	-	-	-	-	-	-	-	-	+	-	-	-	-	-	-	-	-	-	+	-	-
Growth 45°C	-	-	-	-	-	-	-	-	-	-	-	+	-	+	+	-	-	-	-	-	-	+	+	+	-	-
Growth 4.5% NaCl	-	-	-	-	-	+	-	-	-	-	+	+	-	-	-	-	-	+	+	+	-	+	-	-	+	-
Growth 7.0% NaCl	-	-	-	-	-	-	-	-	-	+	+	-	-	-	-	-	-	-	-	-	-	-	-	-	-	-
Growth pH 4.5	+	+	+	+	+	-	-	-	+	-	+	+	+	+	+	+	+	+	-	+	+	+	+	+	-	+

^a^The strain names correspond with the list showed on Table 1. +, good growth; -, no growh; d, poor growth. All strains fermented D-glucose and sucrose. No strains fermented L-sorbose, rhamnose, inositol, sorbitol, inulin, melezitose, xylitol, fucose, arabitol or gluconate.

### DNA-DNA hybridization experiments confirmed the separate status of *Lactobacillus taiwanensis *strains at the species level

DNA similarity values were determined by DNA-DNA hybridization with 26 *Lactobacillus *strains. DNA of the 26 strains was immobilized in membrane blots and the genomic DNA of six *Lactobacillus *type strains and *L. taiwanensis *BL263 strain was used as probe (Table 4). Relative hybridization values obtained from intra-species hybridization assays with *L. intestinalis*, *L. acidophilus *or *L. crispatus *strains always rendered values above 82%, while inter-species DNA re-association values between those species were low (1% to 18%). Results obtained with *L. johnsonii*, *L. gasseri *and *L. taiwanensis *were less clear-cut. *L. johnsonii *and *L. gasseri *intra-species hybridization values were greater than 71%, but *L. johnsonii *BL261 and *L. gasseri *BL223 only showed values of 57% and 62% with their respective type strains. Nevertheless, those values still fall within relaxed species delineation boundaries (50-70% DNA reassociation) [20]. *L. taiwanensis *showed intra-specific hybridization values in the range of 73% to 95%. Inter-species hybridizations between *L. johnsonii *and *L. gasseri *strains rendered values up to 46%, and always higher than with the other species included in this assay, supporting that they are closely related but distinct species. *L. taiwanensis *strains also showed high reassociation rates with *L. johnsonii *and *L. gasseri*, their inter-species hybridization values ranging from 21-40% and from 10-26%, respectively. Therefore, these results also demonstrated the separate status of *L. taiwanensis *strains at the species level, and undoubtedly placed them within the *L. acidophilus *complex. Inside this group they showed closer DNA homology to *L. johnsonii *and *L. gasseri *than to *L. crispatus *and *L. acidophilus *in agreement with the previous phylogenetic analyses.

**Table 4:** DNA re-association values as determined by DNA macroarray analysis among *Lactobacillus *strains

	*L. intestinalis *DSMZ 6629^T^	*L. johnsonii *ATCC 33200^T^	*L. crispatus *DSMZ 20584^T^	*L. gasseri *ATCC 33323^T^	*L. acidophilus *CECT 903^T^	*L. taiwanensis *BL263
*L. murinus *BL262	1.95 ± 1.39	3.68 ± 2.29	2.41 ± 0.83	4.82 ± 2.47	1.23 ± 0.07	15.22 ± 4.65
*L. reuteri *BL259	4.33 ± 4.16	6.04 ± 4.61	3.87 ± 4.17	8.12 ± 4.71	5.93 ± 2.16	4.78 ± 2.07
*L. intestinalis *DSMZ 6629^T^	**100.00**	6.00 ± 1.26	3.66 ± 1.10	4.08 ± 0.86	6.79 ± 2.11	3.52 ± 1.05
*L. intestinalis *BL260	**107.55 ± 4.18**	9.90 ± 3.70	7.75 ± 4.33	9.93 ± 4.83	9.77 ± 0.23	7.27 ± 1.86
*L. johnsonii *ATCC 33200^T^	7.74 ± 1.49	**100.00**	5.15 ± 0.70	19.32 ± 1.51	7.57 ± 1.73	20.76 ± 1.32
*L. johnsonii *CECT 289	10.44 ± 3.74	**81.91 ± 8.39**	5.45 ± 2.02	23.12 ± 7.98	8.61 ± 3.24	28.28 ± 6.53
*L. johnsonii *NCC 2822	15.39 ± 8.09	**94.25 ± 12.15**	1.39 ± 6.29	45.64 ± 4.48	13.81 ± 6.55	35.37 ± 11.93
*L. johnsonii *NCC 1741	17.57 ± 7.53	**91.82 ± 8.88**	10.48 ± 6.32	38.06 ± 8.11	10.39 ± 3.73	35.18 ± 9.31
*L. johnsonii *NCC 533	9.70 ± 5.52	**76.78 ± 8.09**	9.73 ± 7.20	24.53 ± 8.96	8.31 ± 3.82	24.94 ± 8.71
*L. johnsonii *NCC 2767	16.00 ± 7.28	**106.05 ± 7.91**	13.33 ± 8.54	45.02 ± 14.95	11.35 ± 3.62	39.75 ± 11.45
*L. johnsonii *BL261	7.07 ± 3.58	**56.49 ± 8.70**	6.04 ± 2.44	19.74 ± 3.73	12.02 ± 10.84	24.15 ± 2.27
*L. crispatus *DSMZ 20584^T^	7.34 ± 2.86	6.22 ± 1.06	**100.00**	4.28 ± 2.06	11.70 ± 5.35	4.20 ± 1.90
*L. crispatus *BL221	7.14 ± 2.66	6.84 ± 0.86	**82.06 ± 10.06**	4.88 ± 2.97	8.40 ± 2.87	4.21 ± 1.34
*L. gasseri *ATCC 33323^T^	3.99 ± 1.88	15.62 ± 2.19	3.63 ± 1.61	**100.00**	3.25 ± 0.77	9.89 ± 2.56
*L. gasseri *NCC 2856	10.56 ± 5.92	31.63 ± 9.46	7.73 ± 3.08	**76.31 ± 6.36**	6.57 ± 2.36	21.74 ± 6.62
*L. gasseri *NCC 2857	11.54 ± 6.36	41.76 ± 12.27	11.11 ± 6.48	**79.66 ± 15.14**	8.73 ± 3.21	25.55 ± 8.95
*L. gasseri *NCC 2858	8.07 ± 3.82	29.36 ± 9.15	5.98 ± 3.43	**71.09 ± 5.46**	8.92 ± 4.47	19.56 ± 5.14
*L. gasseri *BL223	5.06 ± 2.07	21.28 ± 1.75	4.21 ± 1.38	**62.11 ± 9.80**	3.29 ± 0.88	14.13 ± 2.67
*L. acidophilus *CECT 903^T^	8.28 ± 2.65	7.06 ± 0.08	9.84 ± 3.14	6.18 ± 2.42	**100.00**	4.74 ± 0.80
*L. acidophilus *CECT 4529	8.59 ± 3.38	6.79 ± 1.73	9.08 ± 3.40	3.68 ± 0.61	**98.60 ± 6.52**	3.96 ± 1.23
*L. acidophilus *CECT 4179	7.10 ± 3.61	5.12 ± 1.50	6.72 ± 2.72	3.39 ± 1.61	**101.10 ± 2.45**	3.96 ± 0.64
*L. taiwanensis *BL263	7.50 ± 1.18	31.43 ± 6.28	6.98 ± 2.42	22.05 ± 3.10	6.19 ± 1.73	**100.00**
*L. taiwanensis *BL301	3.55 ± 0.51	18.20 ± 5.85	3.82 ± 0.34	12.61 ± 2.42	3.26 ± 0.66	**72.61 ± 10.45**
*L. taiwanensis *BL302	3.57 ± 1.55	19.56 ± 4.20	3.94 ± 0.88	13.18 ± 0.71	2.56 ± 0.89	**94.95 ± 5.69**
*L. taiwanensis *BL303	3.55 ± 0.29	17.65 ± 6.63	3.74 ± 1.00	12.96 ± 2.37	3.23 ± 0.05	**86.54 ± 8.38**
*L. taiwanensis *BL304	3.40 ± 0.25	17.31 ± 6.16	3.47 ± 0.75	12.73 ± 2.10	3.57 ± 0.64	**75.04 ± 8.82**

Values were calculated as percentage of hybridization relative taking as a 100% the hybridization of each strain with itself and they are the means ± SD from three independent hybridization experiments. Intra-specific DNA hybridization values are shown in bold.

### Complete Genome Hybridization between *L. taiwanensis *BL263 and *L. johnsonii *NCC533 using DNA microarrays

**Figure 5:**
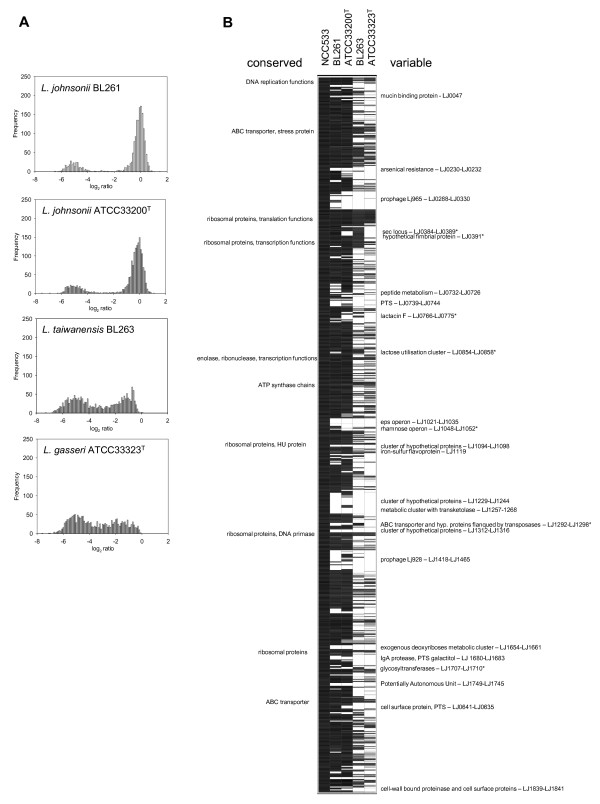
**Comparative Genomic Hybridization (CGH) data**. (A) Frequency distribution histograms of the CGH data. The reference strain is *L. johnsonii *NCC533. Ratios are expressed in a log_2 _scale; (B) CGH data mapped on *L. johnsonii *NCC533. Each horizontal row corresponds to a specific coding region on the array and the genes are vertically ordered according to their positions on the *L. johnsonii *NCC533 genome. The columns represent the strains analyzed, identified by their code numbers. The colour-code gradient ranges from black (presence of a homologous gene) to white (divergence or absence of a gene). Some relevant genes are shown on the left (conserved) or right (variable) alongside the genome. The asterisk showed conserved genes in *L. taiwanensis*, which are optional in *L. johnsonii*.

Firstly, the DNA microarray constructed with oligonucleotides representing all the coding regions from *L. johnsonii *strain NCC533 was tested in a self-hybridization experiment to validate the design of the microarrays. This step was necessary since the microarrays previously used for inter-species CGH in the *L. acidophilus *group [26] were amplicon-based and not oligonucleotide-based. Thus, *L. johnsonii *NCC533 strain was used as both the reference and the test strain, and as expected the distribution of the log_2 _ratios showed a normal distribution around zero (data not shown). The performance of the oligonucleotide microarray was further evaluated by hybridization with DNA from *L. johnsonii *ATCC 33200 type strain and *L. johnsonii *BL261. In the DNA-DNA hybridization assays, this latter strain fell into the relaxed definition of species. The distribution of the log_2 _ratios of the CGH results are shown in Fig. 5A. Approximately 83% of the ORFs present in NCC533 strain produced a log_2 _ratio of -3.5 or greater with these two strains, suggesting a majority of very similar DNA sequences. The rest of ORFs showed log_2 _values under -3.5, reflecting high divergence or absent genes in the test strains. The two *L. johnsonii *strains profiles were typical of intra-species comparisons. In contrast, *L. taiwanensis *and *L. gasseri *strains showed a significant deviation from zero for nearly all the genes, suggesting global sequence divergence between *L. johnsonii *and both tested strains (Fig. 5A). These strains gave a similar profile, characteristic of inter-species comparisons. However, *L. taiwanensis *have more probes (51%) with ratios of -3.5 or greater than *L. gasseri *(47%), showing slightly better gene conservation. This is in agreement with the results of DNA-DNA hybridization experiments. These results confirmed the separate status of *L. taiwanensis *from *L. johnsonii *at the level of species, and they showed that *L. taiwanensis *is closer to *L. johnsonii *than *L. gasseri *is to *L. johnsonii*.

CGH results were mapped on the *L. johnsonii *NCC533 genome (Fig. 5B). The genetic differences observed in the intra-species analysis with the *L. johnsonii *ATCC 33200 type strain closely match with the previous analysis by amplicon-based microarrays [26], further validating the oligonucleotide-based microarrays used in this study. While *L. taiwanensis *BL263 shared fewer genes with the reference *L. johnsonii *NCC533 than the other *L. johnsonii *strains, it showed 78 genes which are not always present in *L. johnsonii *strains. They are mainly organized in 6 gene clusters: the *sec *locus, a hypothetical fimbrial protein, the bacteriocin lactacin F biosynthesis operon, the lactose utilization cluster, the sugar nucleotide dTDP-rhamnose synthesis operon and glycosyltransferases (Fig. 5B, marked with an asterisk). Notably, the most similar genes, thus showing the highest log_2 _ratios (≥ -0.34) for a complete cluster, between *L. johnsonii *and *L. taiwanensis *form a clear-cut cluster containing an ABC transporter and hypothetical proteins, which is flanked by transposases (LJ1292-LJ1298). This cluster is absent in all other strains of *L. johnsonii *and *L. gasseri *analyzed to date. Taken together, these observations strongly argue for an horizontal gene transfer. Considering the marked conservation of this cluster compared to the rest of the *L. taiwanensis *genome, the hypothesis of its loss in the rest of the analysed strains is very unlikely.

**Figure 6:**
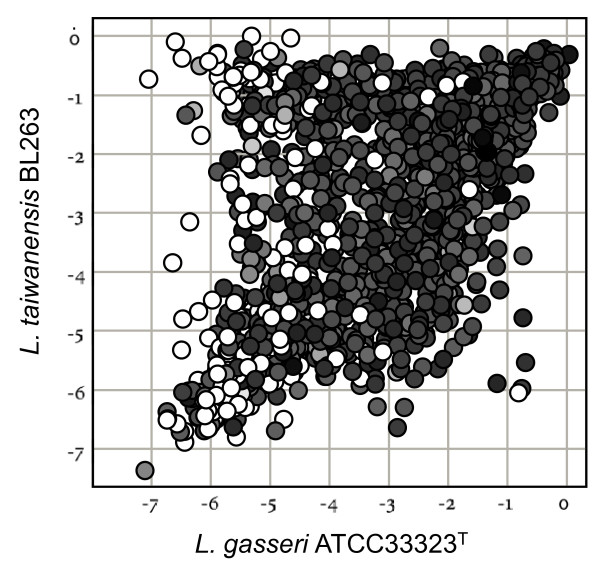
**Genome conservation on *L. johnsonii *NCC533 microarrays**. Scatter plot diagram of hybridization profiles for *L. taiwanensis *BL263 (y axis) versus *L. gasseri *ATCC 33323^T ^(x axis). The axis values represent the hybridization signal ratios expressed in a log_2 _scale. For each gene, data points were colour-coded according to the hybridization profile of a third strain, *L johnsonii *ATCC 33200^T^. Gene conservation colour was obtained from the signal ratio for each gene in log_2 _scale. It ranged from black (presence in ATCC 32200^T ^of a homologous gene with respect to the reference strain NCC533) to white (absence of a gene). For instance, a white circle in the top left corner of the plot represents a gene present in BL263 and absent in both ATCC 33323^T ^and ATCC 32200^T^.

In order to compare the genome conservation of *L. taiwanensis *BL263, *L. gasseri *ATCC 33323^T ^and *L. johnsonii *ATCC 33200^T ^versus *L. johnsonii *NCC533, their CGH results were combined in Fig. 6. Interestingly, variable or absent genes in *L. taiwanensis *and *L. gasseri *were not always shared by the two species. These "species-related" variable genes counted for almost one quarter of the *L. johnsonii *NCC533 genome. Since they were in minority related to mobile elements, this observation strongly supports three evolutionary branches. The number of genes conserved in *L. taiwanensis *and absent/divergent in *L. gasseri *was 257, whereas those conserved in *L. gasseri *and absent/divergent in *L. taiwanensis *were only 186. These results clearly showed that *L. taiwanensis *is closer to *L. johnsonii *than *L. gasseri *is to *L. johnsonii*. The colour code of the Fig. 6 shows that most of the *L. johnsonii *ATCC 33200^T ^absent/variable genes are also absent in the two other tested species. However, 63 genes absent in the *L. johnsonii *type strain are present in *L. taiwanensis *(Additional file 1). In contrast, a similar analysis performed with the CGH results of *L. johnsonii *BL261 instead of *L. johnsonii *ATCC 33200^T ^retrieves only 34 genes absent in *L. johnsonii *BL261 and present in *L. taiwanensis *(Additional file 1). This difference may reflect the adaptation to different ecological niches, since *L. johnsonii *type strain have been isolated from human blood, whereas *L. johnsonii *strains NCC533 and BL261 and *L. taiwanensis *strain BL263 were isolated from intestinal content. Several genes conserved in *L. taiwanensis *BL263 and *L. johnsonii *BL261, but not in *L. johnsonii *ATCC 33200^T^, would help to survival in such highly populated and competitive microbial environment. Among those genes are the bacteriocin lactacin F gene cluster, which is flanked by components of the *L. johnsonii *NCC533 mobilome, and genes encoding proteins hypothetically involved in transport and metabolism of carbohydrates (cellobiose-specific PTS, trehalose-specific PTS system).

## Discussion

A previous study designed to analyze the prebiotic effect of sorbitol in a rat model, resulted in the isolation of five *Lactobacillus *strains from the intestinal content that could not be assigned conclusively to known species [29]. Here, we have shown that the five isolates share almost identical 16S rRNA gene sequences, they form a tight cluster in the *recA*, *pheS*, *pyrG *and *tuf *phylogenetic reconstructions and DNA-DNA hybridization experiments revealed close relatedness between them at the genomic level (> 72%). They were therefore regarded to belong to the same species. During the preparation of this manuscript a new *L. taiwanensis *species isolated from silage was described [39]. 16S rRNA encoding gene and multilocus sequence analyses showed that our strains isolated from rat intestine belong to *L. taiwanensis *species. This species is placed within the *L. acidophilus *group, but they constituted a different cluster from their closest relatives, *L. johnsonii *and *L. gasseri*. The five *L. taiwanensis *strains have been isolated from the same environment and showed very high homology in the phylogenetic analyses; however, the carbohydrate utilization and growth profiles clearly distinguish *L. taiwanensis *BL301 from the groups made by *L. taiwanensis *strains BL304 and BL302 and by *L. taiwanensis *strains BL303 and BL263 (Table 3 and Fig. 1A). These two couples of strains can be distinguished by their protein profiles (Fig. 1B) and by DNA-DNA hybridization experiments (Table 4). Taken together, these results indicate that the five *L. taiwanensis *isolates constitute distinct strains.

The microarray-based CGH has been used to characterize bacterial intra-species genetic diversity at the whole-genome level [41-46]. It has also been used for genome comparisons between species of the same genus or closely related genus that differ in environmental origin or virulence potential [47-49]. CGH analyses by microarrays have also been used to discriminate between the species of the *L. acidophilus *complex [26]. Here, microarray-based CGH technology has been applied to characterize a novel species by determining the gene by gene boundaries with its nearest phylogenetic neighbour. The rapid accumulation in the last years of complete genome bacterial sequences (at present there are 721 complete or in progress deposited genomes in the phylum *Firmicutes*, 57 of them of the genus *Lactobacillus*, at the National Center for Biotechnology Information), and the general trend towards an increase in genome sequencing projects will offer the opportunity of applying the DNA microarray-based CGH as an alternative to the classical whole genome DNA macroarray hybridizations for bacterial species determination. At a more affordable price than full genome sequencing, the CGH analysis renders many more information parameters than whole DNA re-association. Although the CGH microarrays presented the limitation of be unable to detect novel genes, it offers the re-association ratio for each ORF, the plot of the signal to ratio frequency, and the mapping of the conserved genes on the reference genome, thus providing a different ground for intra and inter-species comparisons. This work has shown that the inter-species profile comparison of the novel species *L. taiwanensis *clearly demonstrated its separate status respect to *L. johnsonii*.

The values obtained when comparing the relatedness of *L. taiwanensis *BL263 and *L. johnsonii *NCC533 using DNA-DNA hybridization with macro or microarrays may look dissimilar: whole DNA re-association values were 25% for the macroarray method, but in microarray CGH analysis 51% of the *L. johnsonii *NCC533 genes were conserved in *L. taiwanensis*. In this latter experiment, the hybridization ratios distribution of the conserved genes in *L. taiwanensis *is centred on -1 (Fig. 5A), which corresponds to a 50% reduced signal intensity. Therefore, 51% of conserved genes showing about 50% of signal intensity approximates 25% of global intensity, as observed by DNA-DNA hybridization.

A number of features on *L. johnsonii *NCC533 genome [13] that are conserved on *L. taiwanensis *BL263 may contribute to the adaptation of this bacterium to its ecological niche. Interestingly, our combined CGH results with two *L. johnsonii *strains showed that *L. taiwanensis *BL263 is closer to the gut isolate *L. johnsonii *BL261 than to the blood isolate *L. johnsonii *ATCC 33200^T^. The *L. johnsonii *NCC533 genome encodes 16 putative phosphoenolpyruvate: sugar phosphotransferase systems (PTSs) and several of them, including the ones annotated for fructose, glucose and cellobiose transport, are specifically induced in the gastrointestinal tract (GIT) [14]. CGH results showed that four PTSs, hypothetically involved in transport and metabolism of fructose, cellobiose, trehalose and sucrose are conserved in *L. taiwanensis *BL263. These predictions are supported by the API 50CH results showed for *L. taiwanensis *BL263 (Table 3). A putative maltose/maltodextrin utilization gene cluster, which includes a gene coding for a putative neopullulanase/maltogenic α-amylase, is conserved in *L. taiwanensis *BL263, suggesting that this strain may use starch-degraded products. Other enzymes that may contribute to the survival/persistence of *L. taiwanensis *BL263 in the GIT are bile salt hydrolases (BSH). These enzymes have been almost exclusively found in bacterial species associated with the GIT and its role has been largely discussed [see review [50]. *L. taiwanensis *BL263 exhibited taurocholic and taurodeoxycholic acid deconjugation activity (L. A. Sarmiento-Rubiano and M. J. Yebra, unpublished results). This is in agreement with the CGH analysis showing that a putative operon encoding a BSH and two bile salt transporters (LJ0056 to LJ0058) on *L. johnsonii *NCC533 genome is conserved in *L. taiwanensis *BL263, although this strain has only one of the two transporter encoding genes. Homologues of these genes in *L. johnsonii *100-100 were shown to be gene duplicates and to have a function in taurocholic acid uptake [51,52]. The ability of *L. johnsonii *NCC533 to interact with mucins and epithelial cells has been shown to likely rely on cell-surface associated elongation factor Tu and heat shock protein GroEL [53,54]. As expected for these classes of proteins, their encoding genes (LJ1009 and LJ0461) are also conserved on *L. taiwanensis *BL263, offering a putative role of the encoded proteins in the interaction of this strain with the host. This interaction may also be influenced by the presence of another conserved gene encoding a putative adhesin (LJ0391) that showed similarities with Fap1 fimbrial protein from *Streptococcus parasanguinis *[55].

Previous studies using DNA microarray-based CGH and *in silico *comparative genomic analysis showed a wide sequence similarity and an accurate genome synteny between *L. johnsonii *and *L. gasseri *species [13,26]. In spite of this tight relationship, the novel *Lactobacillus *species described here is placed in a third branching of similar closeness, which raised once more the question about the limits of species delineation. Additionally, the CGH analysis showed that the new species is genetically slightly closer to *L. johnsonii *than *L. gasseri *is. This result is also supported by our DNA-DNA hybridization data and our phylogenetic analysis of *recA*, *pheS*, *pyrG *and *tuf *sequences in these three species.

## Conclusion

Since the genome is the final target of all the molecular taxonomic indicators of species determination, the DNA microarray-based CGH analysis is the most powerful technology for strain typing and species assignment, just behind the costly genome sequencing. In this work we have characterized with conventional taxonomic analyses a novel *Lactobacillus *species within the *L. acidophilus *group, and with microarray-based CGH analysis we confirmed the status of *L. taiwanensis *BL263 as species showing gene by gene differences and similarities with its closest relative *L. johnsonii *strain NCC533.

## Competing interests

The authors declare that they have no competing interests.

## Authors' contributions

LASR has isolated the five strains of the *L. taiwanensis *species and performed all the experiments related to conventional taxonomic analyses. BB designed and analyzed the microarray-based CGH experiments. DM performed the microarray-based CGH experiments. MZ designed and performed the phylogenetic analyses. GPM and MJY designed the study and supervised it. MJY drafted the manuscript. LSAR, BB, MZ and GPM helped improving the draft. All authors read and approved the final manuscript.

## Supplementary Material

Additional file 1 **Genes on *L. johnsonii *NCC533 that are conserved on *L. taiwanensis *BL263 but not in *L. johnsonii *ATCC 33200^T ^or *L. johnsonii *BL261**. This table contains 63 and 34 genes on *L. johnsonii *NCC533 that are conserved on *L. taiwanensis *BL263 and absence on *L. johnsonii *type strain or *L. johnsonii *BL261, respectively. Click here for file
