# Supplementary material for: Characterization of a novel Lactobacillus species closely related to Lactobacillus johnsonii using a combination of molecular and comparative genomics methods
Source: BMC Genomics. 2010 Sep 17;11:504. doi: 10.1186/1471-2164-11-504 (PMC2997000; doi:10.1186/1471-2164-11-504)
Supplement: Additional file 1 — Genes on L. johnsonii NCC533 that are conserved on L. taiwanensis BL263 but not in L. johnsonii ATCC 33200T or L. johnsonii BL261. This table contains 63 and 34 genes on L. johnsonii NCC533 that are conserved on L. taiwanensis BL263 and absence on L. johnsonii type strain or L. johnsonii BL261, respectively. [file 1471-2164-11-504-S1.DOC]

| **Genes conserved on *L. taiwanensis* BL263 but not in *L. johnsonii* ATCC 33200^T^** | |  |
| --- | --- | --- |
| LJ0026 | ABC transporter ATP-binding protein | |
| LJ0027 | Transcriptional regulator, Xre family | |
| LJ0184 | PTS system, cellobiose-specific IIC component | |
| LJ0243 | Hypothetical protein | |
| LJ0275 | Transposase of mobile element | |
| LJ0384 | Protein translocase subunit secY | |
| LJ0385 | Hypothetical protein | |
| LJ0386 | Hypothetical protein | |
| LJ0387 | Hypothetical protein | |
| LJ0388 | Protein translocase subunit secA | |
| LJ0389 | Probable poly(Glycerol-phosphate) alpha-glucosyltransferase (EC 2.4.1.52) | |
| LJ0390 | Surface protein Pls | |
| LJ0391 | Hypothetical protein | |
| LJ0393 | Glycosyltransferase involved in cell wall biogenesis (EC 2.4.-.-) | |
| LJ0394 | Nucleotide sugar synthetase | |
| LJ0600 | ATP-binding transport protein natA | |
| LJ0601 | Hypothetical protein | |
| LJ0602 | Hypothetical protein | |
| LJ0621 | Cell surface protein | |
| LJ0623 | Beta-glucoside operon antiterminator | |
| LJ0624 | PTS system, beta-glucoside-specific IIABC component (EC 2.7.1.69) | |
| LJ0711 | Hypothetical protein | |
| LJ0712 | Phosphoglycerate mutase (EC 5.4.2.1) | |
| LJ0718 | Dipeptidase (EC 3.4.-.-) | |
| LJ0719 | Aminopeptidase C (EC 3.4.22.40) | |
| LJ0758 | Trehalose-6-phosphate hydrolase (EC 3.2.1.93) | |
| LJ0759 | Trehalose operon transcriptional repressor | |
| LJ0760 | PTS system, trehalose-specific IIBC component (EC 2.7.1.69) | |
| LJ0766 | Transcriptional regulatory protein | |
| LJ0767 | Bacteriocin processing peptidase / Bacteriocin export ABC transporter | |
| LJ0768 | Hypothetical protein | |
| LJ0769 | Bacteriocin | |
| LJ0769b | Hypothetical protein | |
| LJ0770 | Hypothetical protein | |
| LJ0771 | Hypothetical protein | |
| LJ0774 | Bacteriocin processing peptidase / Bacteriocin export ABC transporter | |
| LJ1018 | FrnE protein | |
| LJ1020 | Hypothetical protein | |
| LJ1022 | Chain length regulator (capsular polysaccharide biosynthesis) | |
| LJ1049 | dTDP-glucose 4,6-dehydratase (EC 4.2.1.46) | |
| LJ1050 | Glucose-1-phosphate thymidylyltransferase (EC 2.7.7.24) | |
| LJ1051 | dTDP-4-dehydrorhamnose 3,5-epimerase (EC 5.1.3.13) | |
| LJ1122 | ABC transporter ATP-binding protein uup | |
| LJ1128 | Erythrocyte membrane binding protein | |
| LJ1152 | Hypothetical protein | |
| LJ1161 | Proline rich protein precursor | |
| LJ1162 | Hypothetical protein | |
| LJ1235 | Diadenosine 5',5'''-P1,P4-tetraphosphate (Ap4A) asymmetrical hydrolase, other putative hydrolasesof the HIT family and galactose-1-phosphate uridylyltransferase | |
| LJ1237 | Hypothetical protein | |
| LJ1293 | Hypothetical protein | |

**Additional file 1**. Genes on *L. johnsonii* NCC533 that are conserved on *L. taiwanensis* BL263 but not in *L. johnsonii* ATCC 33200^T^ or *L. johnsonii* BL261.

| LJ1294 | Hypothetical protein | |
| --- | --- | --- |
| LJ1295 | Hypothetical protein | |
| LJ1296 | ABC transporter ATP-binding protein | |
| LJ1297 | Transcriptional regulator, Xre family | |
| LJ1458 | Mobile element. Phage protein | |
| LJ1707 | Glycosyl transferase, family 8 / Hypothetical protein | |
| LJ1708 | Glycosyl transferase, family 8 / Hypothetical protein | |
| LJ1709 | Hypothetical protein | |
| LJ1710 | Hypothetical protein | |
| LJ1711 | Hypothetical protein | |
| LJ1730 | ABC transporter ATP-binding protein | |
| LJ1802 | Hypothetical protein | |
| LJ1841 | Peptidyl-prolyl cis-trans isomerase (EC 5.2.1.8) | |
|  |  | |
| **Genes conserved on *L. taiwanensis* BL263 but not in *L. johnsonii* BL261** | |  |
| LJ0420 | Transcriptional regulator, Xre family | |
| LJ0421 | Hypothetical protein | |
| LJ0440 | Chloride channel protein | |
| LJ0523 | Multidrug resistance protein B | |
| LJ0524 | Multidrug resistance protein B | |
| LJ0600 | ATP-binding transport protein natA | |
| LJ0601 | Hypothetical protein | |
| LJ0602 | Hypothetical protein | |
| LJ0624 | PTS system, beta-glucoside-specific IIABC component (EC 2.7.1.69) | |
| LJ0712 | Phosphoglycerate mutase (EC 5.4.2.1) | |
| LJ0860 | Galactose-1-phosphate uridylyltransferase (EC 2.7.7.10) | |
| LJ0861 | Aldose 1-epimerase (EC 5.1.3.3) | |
| LJ1124 | Acetolactate synthase, catabolic (EC 4.1.3.18) | |
| LJ1125 | Alpha-acetolactate decarboxylase (EC 4.1.1.5) | |
| LJ1126 | Hypothetical protein | |
| LJ1235 | Diadenosine 5',5'''-P1,P4-tetraphosphate (Ap4A) asymmetrical hydrolase, other putative hydrolases of the HIT family and galactose-1-phosphate uridylyltransferase | |
| LJ1237 | Hypothetical protein | |
| LJ1292 | Transposase of mobile element | |
| LJ1293 | Hypothetical protein | |
| LJ1294 | Hypothetical protein | |
| LJ1295 | Hypothetical protein | |
| LJ1296 | ABC transporter ATP-binding protein | |
| LJ1297 | Transcriptional regulator, Xre family | |
| LJ1298 | Transposase of mobile element | |
| LJ1425 | Mobile element. Phage protein | |
| LJ1425c | Mobile element. Phage protein | |
| LJ1432 | Mobile element. Phage protein | |
| LJ1433 | Mobile element. Phage protein | |
| LJ1458 | Mobile element. Phage protein | |
| LJ1703 | Cobalt transport protein cbiQ | |
| LJ1709 | Hypothetical protein | |
| LJ1710 | Hypothetical protein | |
| LJ1730 | ABC transporter ATP-binding protein | |
| LJ1841 | Peptidyl-prolyl cis-trans isomerase (EC 5.2.1.8) | |
